# Characterization of the 172 SNPs Included in the ForenSeq™ DNA Signature Prep Kit in a Population from Northeast Italy

**DOI:** 10.3390/ijms26115035

**Published:** 2025-05-23

**Authors:** Chiara Saccardo, Domenico De Leo, Stefania Turrina

**Affiliations:** Department of Diagnostics and Public Health, Section of Forensic Medicine, University of Verona, P.le L.A. Scuro 10, 37134 Verona, Italy; domenico.deleo@univr.it (D.D.L.); stefania.turrina@univr.it (S.T.)

**Keywords:** forensic genetics, massively parallel sequencing, single nucleotide polymorphisms, microhaplotypes, MiSeq™ FGx Forensic Genomics System

## Abstract

In this study, 172 Single-Nucleotide Polymorphisms (SNPs) (94 identity-informative SNPs, 56 ancestry-informative SNPs, and 22 phenotypic-informative SNPs) included in the ForenSeq™ DNA Signature Prep kit/DNA Primer Mix B (Verogen) were used for genotyping DNA samples from a population of twenty-one unrelated subjects, native to Northeast Italy. SNP sequencing was performed with the MiSeq FGx™ Forensic Genomics System (Illumina-Verogen), and data were analyzed using the Universal Analysis Software (UAS) v1.2. Raw data underwent further examination with STRait Razor v3 (SRv3) to compare the target SNPs’ genotype calls made with UAS and to identify the presence of microhaplotypes (MHs) due to SNPs associated with the same target SNP’s amplicon. The allele (haplotype) frequencies, Hardy–Weinberg equilibrium, linkage disequilibrium, number of effective alleles (A_e_), and relevant forensic statistic parameters were calculated. Among the 172 SNPs evaluated, 45 unique microhaplotypes were found, comprising a novel sequence variant never previously described. The presence of MHs resulted in an 8.00% rise in the typologies of unique sequences, leading to changes in A_e_. Notably, for 12 out of the 94 iiSNPs, the values of A_e_ exceeded 2.00, which is generally associated with a higher expected heterozygosity and increased power of discrimination.

## 1. Introduction

Massively Parallel Sequencing (MPS) is a high-throughput technique that performs both length-based and sequence-based analysis and allows the typing of a significant number of different genetic markers, such as short tandem repeats (STRs), single-nucleotide polymorphisms (SNPs), and insertion–deletion polymorphisms (indels), in an all-in-one multiplex reaction, thus optimizing the amount of DNA available for the analysis [1,2,3,4].

Moreover, compared to traditional capillary electrophoresis (CE), MPS provides detailed information on allele sequence variations detected within the repetitive core and flanking regions of loci. Therefore, the identification of sequence variations using MPS, regardless of the marker’s type, generally involves the detection of additional alleles that, depending on their frequency within the investigated population, can enhance the marker’s informativeness, positively affecting some relevant forensic parameters, such as the power of discrimination, and allowing a more targeted application of genetic markers in different forensic genetic investigations, thus making the MPS an undeniable resource in forensic genetics.

In particular, when mutations affect STR loci, the MPS technique can be exploited to disclose isometric alleles (i.e., two alleles with the same length but different sequences) in homozygous genotypes, and alleles showing different sequences from the reference one in heterozygous genotypes. When mutations involve SNPs, MPS can disclose a novel type of molecular markers identified as microhaplotype loci (MHs) due to additional SNPs and/or insertion/deletion sequences associated with the target SNP within a single amplicon of less than 200–300 base pairs (bp) in length [5,6,7].

Despite microhaplotypes exhibiting the same characteristics as the target SNPs from which they originate, such as a low mutation rate, lack of stutter artifacts, and production of short-length amplicons, being a combination of multiple SNPs, they convert the biallelic SNP marker into a multiallelic system [8]. Thereby, MHs enhance the SNPs’ informativeness, facilitating the interpretation of complex kinship analyses, mixed DNA profiles, and degraded DNA samples. This makes MHs one of the most promising markers to be used in forensic applications [9,10,11,12,13,14,15,16,17,18].

However, the available data on SNPs is still scarce, especially concerning the Italian population. This study focused on the MPS analysis of SNPs that can provide insights into individual identification, phenotypic traits, and biogeographic origins, information not deducible through the STR analysis alone. DNA samples from 21 individuals native to Northeast Italy were sequenced on the MiSeq FGx™ Forensic Genomics System (Illumina, San Diego, CA, USA) equipped with Universal Analysis Software (UAS), v1.2 [19,20], using the DNA Primer Mix B (DPMB) of the ForenSeq™ DNA Signature Prep kit (FSSP) (Verogen, San Diego, CA, USA) [21]. This kit includes, other than an amelogenin marker and 58 STRs (27 autosomal STRs (auSTRs), 24 Y-STRs, and 7 X-STRs), 172 SNPs subdivided into 94 identity-informative (iiSNPs), 22 phenotypic-informative (piSNPs), and 54 ancestry-informative (aiSNPs) SNPs. After sequences examination, the allele frequency and the number of effective alleles (A_e_) for each target SNP and microhaplotype locus were determined to assess their potential impact on relevant forensic parameters.

## 2. Results and Discussion

Given that both rs16891982 and rs12913832 are suitable for ancestry and phenotype prediction, this study incorporated them into the aiSNPs set.

The expected number of genotypes for the 21 subjects typed with the 172 SNPs from the FSSP kit should be 3612, which aligns with the number of genotypes obtained through UAS genotyping.

Assuming that all investigated SNPs are heterozygous, the maximum number of alleles that could be obtained from genotyping 172 SNPs using UAS is 344. In this study, 327 alleles were identified. The shortfall of 17 alleles was due to monomorphic SNPs detected for eleven aiSNPs (rs2814778, rs1834619, rs3827760, rs3811801, rs3823159, rs1871534, rs3814134, rs671, rs1426654, rs1800414, and rs2042762) and six piSNPs (N29insA, rs11547464, rs1805006, rs1805009, rs201326893_Y152OCH, and rs885479). The finding of a monomorphic state for some aiSNPs and piSNPs is not surprising, considering that aiSNPs and piSNPs have the ability to trace the ancestral origins of a population and reveal the shared phenotypic traits (i.e., eye and hair color) among individuals with common ancestors, thus contributing to the characterization of a population. Moreover, the observation of monomorphic SNPs in this study is consistent with findings from other studies conducted on larger populations [22,23,24], suggesting that the monomorphic SNP state is mainly correlated with the characteristics of the investigated population and, to a lesser degree, with sample size. Allele frequencies for the 172 SNPs included in the DPMB are presented in Table S1.

After the supplementary analysis of the raw FASTQ files using STRait Razor v3 (SRv3) [25], the only discrepancy in genotype calls of target SNPs compared to those made with UAS, when the reads’ thresholds met for both pipelines, was found for the piSNP N29insA, an indel also known as rs86inA and rs312262906 [26], which was genotyped in all 21 subjects using UAS as homozygous for the C allele, while by SRv3 as rs796296176.1Null allele.

Regarding the 22 target piSNPs, SRv3 automatically groups three (rs1805005, rs1805006, and rs2228479) and six (rs1110400, rs11547464, rs1805007, rs1805008, rs201326893_Y152OCH, and rs885479) of them based on their overlapping chromosomal start and end positions, resulting in two distinct amplicons, respectively, named mh16-MC1RB and mh16-MC1RC, which reduce the piSNPs amplicons’ number to 15 [23].

Furthermore, SRv3 found two additional SNPs not included in the FSSP kit, rs755095 and rs12047255. By using the UCSC Genome Browser with the reference genome GRCh38, it was possible to achieve their chromosome locations, ascertaining that rs755095 (Chr21:41044050) is separated from the target iiSNP rs914165 (Chr21:41044003) by 48 bp, while rs12047255 (Chr1:239718578) is 49 bp away from the target iiSNP rs891700 (Chr1:239718626). Therefore, according to the literature [27], these SNPs are located in the flanking region of the two target iiSNPs, forming two microhaplotypes: rs914165-rs755095 and rs891700-rs12047255. However, the genotyping findings of this study reveal that SNP rs755095 consistently presents haplotype C, while rs12047255 always presents haplotype G. As a result, the observed genetic variability is exclusively due to the target iiSNP alleles and is not related to any microhaplotype allele. Therefore, rs755095 and rs12047255 were not further taken into consideration.

The analysis of sequence SNPs’ amplicons allowed the identification of additional SNPs associated with thirty target SNPs (twenty-two iiSNPs, six aiSNPs, and two piSNPs), which, based on the microhaplotype’s definition, can be classified as such.

Considering mh16-MC1RB and mh16-MC1RC, which generated three and four unique microhaplotype alleles, respectively, a total of forty-five unique microhaplotype alleles were identified, as reported in Table S2.

Among the 45 microhaplotype alleles, it is worth noting that a microhaplotype allele has never been previously described, due to the SNP linked to the target aiSNP rs1919550. This DNA sequence mutation was reported by SRv3 as NA, since it is absent in its sequence string-matching database. Therefore, the nomenclature was manually assigned by aligning the sequence reported by SRv3 with the reference sequence GRCh38 from the UCSC Genome Browser. After identifying the G > A mutation at position chr3:121645314, it was possible to associate it with rs190565336, which was further verified using the NCBI SNP database. Moreover, for iiSNP rs4606077 and aiSNP rs7251928, the microhaplotype alleles arose from T > G and C > T mutations at positions chr8:143574562 and chr19:4077087, respectively. For both these mutations, SRv3 was unable to match the dbSNP rs numbers that were subsequently identified in rs72691478 and rs549346879, respectively, by using the National Center for Biotechnology Information (NCBI) SNP database.

Additionally, a new A variant allele for the piSNP rs2402130 was identified, resulting in a microhaplotype characterized by the deletion of a trinucleotide repeat (TCA) that changes the sequence string from [TCA]6 to [TCA]5. Given that this A variant allele differs from the more commonly found one, the allelic designation was assigned manually and reported as A[TCA/-].

Furthermore, for the target piSNP rs1805009, genotyped with UAS as monomorphic, two distinct sequence-strings were revealed when the amplicon sequence analysis was considered. One sequence corresponds to the target SNP allele, while the other one is referable to a microhaplotype allele resulting from an accessory SNP (rs2228478) involving an A > G mutation in the flanking region.

Overall, excluding the two amplicons mh16-MC1RB and mh16-MC1RC, which include 9 piSNPs, the number of distinct haplotypes identified for the 163 SNPs (94 iiSNPs, 56 aiSNPs, and 13 piSNPs) increased from 314 to 340, representing an 8% increase due to the detection of microhaplotypes, including a previously undescribed one.

Twelve microhaplotypes were observed only once, but given the limited number of subjects involved in the present study, they cannot be classified as rare variants (minor allele frequency (MAF) < 1%) [28]. However, from a comparison with those reported in other studies [23,29,30], it was possible to ascertain how these sequences are effectively rare. This suggests that the findings remain relevant in this context despite the small sample size.

The observed (Ho) and expected (He) heterozygosity and Hardy–Weinberg equilibrium (HWE) test, calculated using Arlequin for each target SNP, also comprehensive of MHs, are detailed in Table S3.

Excluding 16 monomorphic target SNPs for which no data were available, for the iiSNPs, He ranged from a maximum value of 0.733 for rs1109037 to a minimum of 0.251 for both rs938283 and rs6955448. For the aiSNPs, He varied from a maximum of 0.535 for rs2024566 to a minimum of 0.048, observed for rs3737576, rs174570, rs12439433, rs4471745, and rs3916235. For the piSNPs, He ranged from a maximum of 0.512 for rs1042602 to a minimum of 0.048 for rs1110400 and rs1805008.

A small number of SNPs (four iiSNPs and one aiSNP) showed a significant deviation from the Hardy–Weinberg equilibrium, with a *p*-value (*p*) lower than 0.05. However, after applying the Bonferroni correction (*p* = 0.000291), all SNPs were found to be in the HWE, with the only exception for the iiSNP rs4606077, which maintained the deviation from the HWE (*p* = 0.00011).

Based on the pairwise linkage disequilibrium (LD) test performed on SNPs located on the same chromosome, 28 pairs of SNPs were identified with exact *p*-values lower than the predefined significance level (*p* = 0.05). Among these pairs, four exhibited highly significant values (*p* = 0.0000 +/− 0.000), which are highlighted in bold red in Table S4. These include a pair of aiSNPs, rs12498138 and rs1919550, located on chromosome 3; two pairs of piSNPs, rs1042602 and rs1393350, and rs2228479 and rs1805009, located on chromosomes 11 and 16, respectively, and a pair of iiSNPs, rs1736442, and rs1024116, located on chromosome 18. The physical distances between the SNPs in these pairs are 95,416 bp, 606 bp, 99,350 bp, and 20,161,885 bp, respectively. These genetic distances are significantly lower than the commonly accepted threshold of 50 centimorgans (cM), corresponding to 50 megabases (Mb), able to ensure likely independent recombination between the loci. Upon analyzing the frequencies of genetic profiles derived from the combinations of genotypes of the SNP pairs in a linkage disequilibrium, it was observed that microhaplotypes can contribute to generating more unique profiles (Table S5).

Moreover, we assessed whether the transition from biallelic target SNPs to microhaplotype markers leads to a significant variation in the effective allele number (Ae) that can influence some forensic parameters, such as gene diversity (GD = He) and discrimination power (PD) (refer to Table S6).

When the target SNP alone was taken into account, only the iiSNP rs445251 showed an Ae value equal to 2.00, with GD = He and PD values of 0.500 and 0.653, respectively. For the remaining 29 target SNPs, the GD = He values range from 0.00 to 4.99, and the PD values range from 0.00 to 0.635. When microhaplotypes were considered, 13 iiSNP amplicons exhibited Ae values equal to or greater than 2.00, with GD = He values ranging from 0.733 to 0.177 and PD values ranging from 0.862 to 0.254. Even if, usually, an increase in Ae value positively affects the GD = He and PD values, more consistent heterozygosity increments were observed for SNPs with Ae values greater than 2.00, reaching a value that exceeded 0.70 when the Ae value was higher than 3.00, as observed for the iiSNP rs1109037. Similarly, more significant increases for PD were generally found when the SNPs had Ae values greater than 2.00, indicating that the loci have become multiallelic. Worthy of attention is the piSNP rs1805009, targeted with UAS as monomorphic but exhibiting two alleles when SRv3 analysis was performed. In this instance, the second allele was a microhaplotype allele that increases the Ae value, determining a significant rise in heterozygosity from 0.00 to 0.215, and improves the power of discrimination from 0.00 to 0.363.

To support these observations and measure whether there is a linear relationship between variations in Ae values and the increments in He and PD values, the Pearson correlation coefficient (r) was calculated. It was assumed that a Pearson correlation’s r value equal to 0.00 indicates the absence of association between the two variables considered (Ae/He and Ae/PD) and that r values in the range between 0.00 and +1.00 and between 0.00 and −1.00 indicate a positive and negative linear correlation, respectively [31] (p. 219).

Regarding the microhaplotypes related to twenty-two iiSNPs’ amplicons, the Ae/He and Ae/PD r values were 0.953923 and 0.855645, and concerning the microhaplotypes related to six aiSNPs’ amplicons, the r values were 0.980046 and 0.989779. These findings indicate a very strong positive linear correlation between variations in Ae values and increases in He and PD, suggesting a dependable relationship. For the microhaplotypes related to four piSNPs’ amplicons (rs1805009, rs2402130, mh16-MC1RB, and mh16-MC1RC), the Ae/He and Ae/PD values were 0.722716 and 0.669479, respectively, indicating a moderate positive correlation with a substantial relationship (as reported in Table S7).

Anyway, since the Ae value depends on the type and frequency of the allele in the population, detecting a microhaplotype that generates a “new allele” does not necessarily significantly affect the forensic parameters examined, especially when it replaces a wild-type allele or when it is rare (<1%).

For instance, in this study, for seven of the thirty-two microhaplotype loci, no changes in Ae values and forensic parameters were observed, as they remain in a biallelic SNP state exhibiting two distinct biallelic genotype patterns.

One of these two biallelic genotype patterns, identified at the amplicons of five iiSNPs (rs430046, rs279844, rs445251, rs4530059, and rs6955) and one aiSNP (rs1079597), was characterized by a wild-type allele and a microhaplotype allele. As a result, the loci remain biallelic, without an increase in the number of effective alleles and without variations in other forensic parameters. This is exemplified by rs430046, where two alleles were identified: the wild-type C allele and the microhaplotype allele derived from two SNPs (rs409820 and rs430044) located in the flanking region of the wild-type T allele, even though the wild-type T allele was never detected in the population under investigation.

The second biallelic genotype pattern was observed only at iiSNP rs727811, for which the only two alleles detected were microhaplotype alleles generated by the presence of rs1390470 in the flanking region of two wild-type target SNP alleles, G and T (the latter were never found in the studied population). Therefore, also in this case, the detection of microhaplotypes did not affect the Ae value and the forensic parameters.

Additionally, 10 of 32 microhaplotype loci led to the generation of three or more haplotypes, determining a change in the number of effective alleles, even though, due to the very low frequency of the microhaplotype alleles in the population investigated, this does not significantly affect the PD values.

Based on these findings, it can be deduced that the sequence variation revealed as a microhaplotype impacts the forensic parameters of the locus the more frequently it is found in the population. This implies the need to investigate how microhaplotype frequencies fluctuate across broad and diverse populations to determine their usefulness in forensics.

On the other hand, identifying a rare or previously undescribed sequence variation in an individual suspected of committing a crime could assume relevant implications in specific forensic investigative contexts, such as DNA mixture deconvolution and genotype resolution [9,12,13,14,15]. The lower the frequency of the detected sequence variation (less than 1%) in the individual’s origin population, the higher the probability of linking the evidence collected at the crime scene to the potential suspect, thus providing undeniable probative value to the DNA evidence.

## 3. Materials and Methods

### 3.1. DNA Samples, Extraction, and Quantification

Twenty-one anonymized buccal cell samples were collected from unrelated adult individuals across three generations, after obtaining their written informed consent and ethical approval from the Research Ethics Committee of the University of Verona (protocol code: CARU-12/2020).

DNA was extracted employing the QIAamp^®^ DNA Mini Kit (Qiagen, Hilden, Germany), according to the manufacturer’s guidelines. Subsequently, DNA samples were quantified with a Qubit^®^ 2.0 Fluorometer using the Qubit^®^ dsDNA HS Assay Kit (Thermo Fisher Scientific Inc., Waltham, MA, USA), and each DNA sample was normalized to a concentration of 1 ng/μL.

### 3.2. Library Preparation and Sequencing

For each of the twenty-one DNA samples, sequencing was carried out employing the DNA Primer Mix B (DPMB) from the ForenSeq™ DNA Signature Prep Kit (FSSP), which includes primers to amplify an amelogenin marker, 27 auSTRs, 24 Y-STRs, 7 X-STRs, and 172 SNPs (94 iiSNPs, 22 piSNPs, and 56 aiSNPs) (Verogen, San Diego, CA, USA). The library preparation was performed following the manufacturer’s recommended procedure to optimize sequencing performance [21], using 2800 M Control DNA (included in the FSSP kit) and nuclease-free water as positive DNA and blank PCR controls, respectively.

Sequencing was performed on a MiSeq FGx™ Sequencing System (Illumina, San Diego, CA, USA) following the manufacturer’s guidelines [19]. Two sequencing library replicates for each sample were carried out to ensure the sequencing data reproducibility.

### 3.3. Data Analysis

The sequencing data generated by the MiSeq FGx System were analyzed using Universal Analysis Software (UAS) v1.2 (Illumina, San Diego, CA, USA), applying default parameters [20]. Only genetic profiles with a total read count of 85,000 reads or more were considered in the analysis. For all three categories of SNPs, the analytical threshold (AT) was set at 1.5%, and the interpretation threshold (IT) was set at 4.5%. These thresholds were used to determine the percentage of the total number of reads per locus and to make genotype calls, employing a minimum depth threshold of at least 30 reads for the homozygous loci and 10 reads for each of the two alleles in heterozygous loci in order to ensure that each called allele was not a fictitious sequence.

The version of UAS 1.2 only analyzes the marker’s target (not allowing the generation of the “*Flanking Region Report*”), thus precluding the possibility of revealing the presence of any sequence variations in the flanking regions of the SNPs and, consequently, of identifying any microhaplotypes (MHs), i.e., short sequence strings (<300 nucleotides in length) with additional SNPs or insertion/deletion sequences in close physical proximity to the target SNP [5,6,7].

To overcome this limitation, the sequencing data were supplemented by analyzing the FASTQ files with the bioinformatic tool STRait Razor v3 (SRv3) [25], setting the same thresholds fixed for UAS (see above).

SRv3, in its default configuration (ForenSeqv1.27.config), contains the target sequence and flanking region files for each SNP included in FSSP, allowing maximization of the recovery of sequence data from the raw output FASTQ files generated using UAS for each sample and, based on the revealed allele or haplotype, identification of biallelic target SNPs (non-variable haplotypes) and microhaplotypes.

The UAS’s target SNP genotype calls were compared to those provided by SRv3.

The allelic designation of MHs was assigned directly by SRv3, except for sequence variations identified in this study that were not included in the SRv3 string-matching database, for which the designations were assigned manually. Briefly, the SRv3 TSV file containing the string with the not-identified sequence variation was exported to an in-house Excel spreadsheet for alignment with the human reference sequence (GRCh38) available in the UCSC Genome Browser. The region of the SNP investigated, including the amplicon start and end positions, was provided by SRv3.

Once the mutation position was identified, it was confirmed by cross-referencing with the National Center for Biotechnology Information (NCBI) SNP database [32].

### 3.4. Statistical Analysis

Using Arlequin software v3.5.2.2 [33], the allele frequencies, the observed (H_o_) and expected heterozygosity (H_e_), the Hardy–Weinberg equilibrium (HWE) (with a number of steps in the Markov chain = 1 × 10^6^ and a number of dememorization steps = 1 × 10^5^), and the pairwise linkage disequilibrium (LD) (using an exact test with a number of permutations = 1000) among SNPs localized on the same chromosome were calculated, considering a *p* value < 0.05 statistically significant.

Moreover, an in-house Excel spreadsheet was used to evaluate the frequency of genetic profiles generated from the pairwise linked SNPs localized on the same chromosome.

Relevant forensic statistical parameters, such as polymorphism information content (PIC), discrimination power (PD), match probability (MP), and exclusion power (PE), were calculated using the statistical package PowerStats v1.2 [34]. To evaluate how the presence of MHs affects the genetic variability of SNPs, the effective allele (A_e_) number was determined as described by Crow and Kimura formulation [35].

Moreover, to assess whether there was a linear correlation between variations in A_e_ and increments in H_e_ and PD values, Pearson’s correlation coefficient (r) was calculated.

## 4. Conclusions

This study, which has examined the presence of sequence variations in the flanking regions of 172 target SNPs included in the ForenSeq™ DNA Signature Prep Kit/DNA Primer Mix B, represents the only work regarding the Italian population to date.

Although this study involved a limited number of subjects (21), which certainly needs to be expanded in the future to provide further support to the presented findings, it confirmed that the microhaplotypes generated by sequence variations associated with target SNPs generally lead to changes in the type and number of effective alleles of the biallelic target SNPs.

It was observed that A_e_ values greater than 2.00, achieved for microhaplotypes capable of generating three or more haplotypes with population frequency exceeding 1%, generally lead to higher H_e_ e PD values, making the microhaplotypes reasonably useful markers in some forensic applications, such as mixture deconvolution [36].

Nevertheless, slight increments in A_e_ values do not always significantly influence forensic parameters, since these are affected not only by the presence of new alleles (i.e., microhaplotypes) but also by the frequency of these alleles within the population.

However, it is undeniable that the possibility of revealing microhaplotypes with MPS provides more detailed information on genetic markers and contributes to improving and enhancing forensic parameters that better characterize populations, thus making MPS an indispensable investigative resource in forensic genetics.

## Data Availability

The data presented in this study are included in the article and in the Supplementary Materials.

## Supplementary Materials

The following supporting information can be downloaded at: https://www.mdpi.com/article/10.3390/ijms26115035/s1.
